# 4335 Role of PSD95 and nNOS interaction in gene regulation following fear conditioning and implications for molecular mechanisms underlying PTSD

**DOI:** 10.1017/cts.2020.90

**Published:** 2020-07-29

**Authors:** Jheel Patel, Erik Dustrude, Melissa Haulcomb, Liangping Li, Guanglong Jiang, Yunlong Liu, Yvonne Lai, Andrei Molosh, Anantha Shekhar

**Affiliations:** 1 Indiana University School of Medicine

## Abstract

OBJECTIVES/GOALS: Normal fear learning produces avoidance behavior that promotes survival, but excessive and persistent fear after trauma can lead to development of phobias and post-traumatic stress disorder (PTSD). Our goal is to understand the mechanism and identify novel genetic targets underlying fear responses. METHODS/STUDY POPULATION: Involvement of the amygdala in fear acquisition is well established and requires activation of N-methyl-D-aspartic acid receptors (NMDARs). At a cellular level, NMDAR activation leads to production of nitric oxide (NO) by a process mediated by interaction between postsynaptic density protein 95 (PSD95) and neuronal nitric oxide synthase (nNOS). To elucidate mechanisms underlying the role of the PSD95-nNOS-NO pathway in conditioned fear, here we use rodent behavioral paradigms, pharmacological treatment with a small molecular PSD95-nNOS inhibitor, co-immunoprecipitation, Western blotting, and RNA-sequencing. RESULTS/ANTICIPATED RESULTS: We show that fear conditioning enhances the PSD95-nNOS interaction and that the small-molecule ZL006 inhibits this interaction. Treatment with ZL006 also attenuates rodent cued-fear consolidation and prevents fear-mediated shifts in glutamatergic receptor and current densities in the basolateral amygdala (BLA). With RNA-sequencing, expression of 516 genes was altered in the BLA following fear expression; of these genes, 83 were restored by systemic ZL006 treatment. Network data and gene ontology enrichment analysis with Ingenuity Pathway Analysis and DAVID software found that cell-cell interaction, cognition-related pathways, and insulin-like growth factor binding were significantly altered. DISCUSSION/SIGNIFICANCE OF IMPACT: Our results reveal novel genetic targets that underlie plasticity of fear-memory circuitry via their contribution of NMDAR-mediated fear consolidation and can inform future strategies for targeting fear related disorders like PTSD. CONFLICT OF INTEREST DESCRIPTION: Anantha Shekhar and Yvonne Lai are co-founders of Anagin, Inc., which is developing some of the related molecules for the treatment of PTSD.

